# Photoelectric H_2_S Sensing Based on Electrospun Hollow CuO-SnO_2_ Nanotubes at Room Temperature

**DOI:** 10.3390/s24196420

**Published:** 2024-10-03

**Authors:** Cheng Zou, Cheng Peng, Xiaopeng She, Mengqing Wang, Bo Peng, Yong Zhou

**Affiliations:** 1 Liangjiang School of Artificial Intelligence of Chongqing University of Technology, Chongqing 401135, China; zouchengidrc@cqu.edu.cn; 2 Key Laboratory of Optoelectronic Technology and System of Ministry of Education, College of Optoelectronic Engineering, Chongqing University, Chongqing 400044, China; 3 Chongqing Key Laboratory of Natural Product Synthesis and Drug Research, Innovative Drug Research Center, School of Pharmaceutical Sciences, Chongqing University, Chongqing 401331, China

**Keywords:** conductometric H_2_S sensors, SnO_2_, heterojunctions, light activation, electrospun nanotubes

## Abstract

Pure tin oxide (SnO_2_) as a typical conductometric hydrogen sulfide (H_2_S) gas-sensing material always suffers from limited sensitivity, elevated operation temperature, and poor selectivity. To overcome these hindrances, in this work, hollow CuO-SnO_2_ nanotubes were successfully electrospun for room-temperature (25 °C) trace H_2_S detection under blue light activation. Among all SnO_2_-based candidates, a pure SnO_2_ sensor showed no signal, even toward 10 ppm, while the 1% CuO-SnO_2_ sensor achieved a limit of detection (LoD) value of 2.5 ppm, a large response of 4.7, and a short response/recovery time of 21/61 s toward 10 ppm H_2_S, as well as nice repeatability, long-term stability, and selectivity. This excellent performance could be ascribed to the one-dimensional (1D) hollow nanostructure, abundant p-n heterojunctions, and the photoelectric effect of the CuO-SnO_2_ nanotubes. The proposed design strategies cater to the demanding requirements of high sensitivity and low power consumption in future application scenarios such as Internet of Things and smart optoelectronic systems.

## 1. Introduction

Conductometric gas sensors have been extensively applied in various fields, such as in the monitoring of agricultural fragrance release [1], industry production leakage [2], indoor ventilation [3], and environmental pollution [4,5,6,7,8]. Of the diverse gas-sensitive materials, semiconducting metal oxides gain the most popularity due to their easy synthesis, low cost, and the rich modulation methodology of their structural and electronic properties. Nevertheless, their elevated operation temperature and severe cross-sensitivity curtail their further development [9]. Take the detection of H_2_S, a colorless, toxic, and flammable gas, as an example. Due to the ubiquity in the petroleum industry, natural gas, and biological decomposition, the short-term (10 min) and long-term (8 h) exposure limits of H_2_S gas for humans are separately 15 and 10 ppm [10,11], while the lowest detection limit of 5 ppm could be achieved by the human olfactory system [12]. Therefore, it is of great necessity to develop a novel gas sensor that can, in real time, monitor trace H_2_S at a several-ppm scale with high sensitivity and excellent selectivity. In this regard, n-type SnO_2_ stands out due to its excellent stability, high sensitivity, and low cost. Unsatisfactorily, pure SnO_2_ sensors readily suffer from high operating temperatures (>200 °C) and weak selectivity [13], thus inducing safety concerns in flammable and explosive application scenarios and frustrating the demands for low power consumption and high miniaturization in future optoelectronic devices. To overcome these obstacles, incorporating other conducting nanofillers including low-dimensional nanomaterials (reduced graphene oxide, carbon nanotubes, MXenes, etc.) into the host SnO_2_ material is a feasible strategy [14,15,16]. However, difficult as it is, ingredient distribution within the composite bulk and phase separation between different categories of components readily occurred, which deteriorated the operational robustness of the as-prepared sensors [17]. Alternatively, prepared nanostructured metal oxides (large surface energy and surface area) or external light irradiation could achieve the same goals [18,19]. In this work, our group first employed electrostatic electrospinning technology and subsequent calcination treatment to prepare 1D CuO-SnO_2_ nanotubes with a mixture of two different metal salt precursors to suppress the phase separation. Such structural features could maximize the Knudsen effect and accelerate the diffusion/adsorption of the target gasses within the sensing layer. Then, visible light irradiation was applied to stimulate the gas–solid reactions. Both nanostructure-related performance modulation and photoelectric effect were simultaneously leveraged and anticipated to realize sensitive H_2_S detection at room temperature.

## 2. Materials and Methods

The primary metal salt precursors copper chloride dihydrate (CuCl_2_·2H_2_O, ≥99%) and tin tetrachloride pentahydrate (SnCl_4_·5H_2_O, ≥99%) were purchased from Shanghai Macklin Biochemical Co., Ltd. (Shanghai, China). Polyvinyl pyrrolidone (PVP, M = 1,300,000) was obtained from Shanghai Aladdin Biochemical Technology Co., Ltd. (Shanghai, China). Anhydrous N, N-dimethylformamide (DMF), and ethanol were purchased from Chron Chemicals (Chengdu, China). The preparation method of sensitive materials occurred as follows. As is typical, 10 mL of DMF and 15 mL of ethanol was first stirred together. Subsequently, 1.54 g of PVP and 2.32 g of SnCl_4_·5H_2_O were dissolved in the mixed solvent with another magnetic stirring. Finally, CuCl_2_·2H_2_O was added at an atomic ratio of Cu over Sn of 0:100, 1:99, 2:98, and 3:97, which were labeled as pure SnO_2_, 1% CuO-SnO_2_, 2% CuO-SnO_2_, and 3% CuO-SnO_2_, respectively. As for pure CuO sensor, the preparation procedure was similar to that of the 1% CuO-SnO_2_ counterpart, except for the addition of SnCl_4_·5H_2_O precursor. The precursor solution was stirred at a speed of 1000 rad/s at room temperature for 0.5 h.

**Figure 1:**
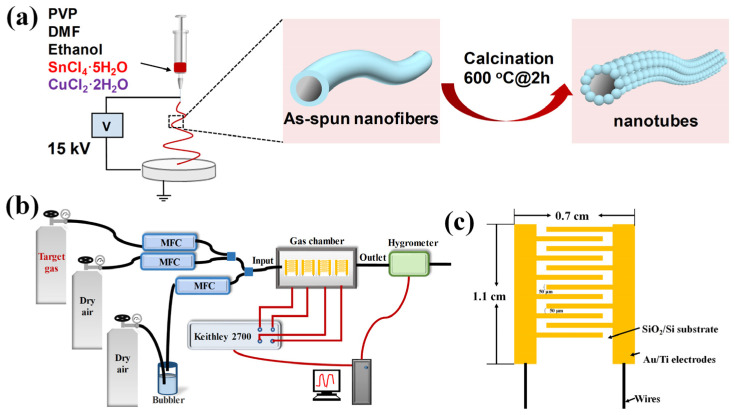
Schematic illustration of (**a**) material preparation, (**b**) test apparatus, and (**c**) IDE device.

Following that, an electrostatic spinning at a high voltage of 15 kV was conducted with a distance of 20 cm between the injector needle and the drum, with a solution injection rate of 1 mL/h, as depicted in Figure 1a. Afterwards, the received fibrous material was dried in a vacuum oven at 80 °C for 6 h, and then heated in a tube furnace at a heating rate of 1 °C/min until 600 °C was reached for 2 h. Then, the temperature was first decreased to 200 °C within 1 h, and then naturally cooled to room temperature to obtain porous CuO-SnO_2_ nanotubes. After this calcination, the powder was dissolved into deionized water and sonicated for 20 min, followed by a drop-coating onto a clean interdigital electrode device (IDE) with both width and interspacing of 50 μm, as shown in Figure 1c. The IDE was then anchored in a seamless gas chamber for the future gas-sensing tests.

During the dynamic tests, the total gas flow was maintained at 1000 mL/min with dry synthesized air as the background atmosphere. The gas concentration was regulated by mass flow controllers (MFCs), as illustrated in Figure 1b. Detailed test procedures and characterization techniques are discussed in Supplementary Material.

## 3. Results and Discussion

### 3.1. Structural and Morphological Characteristics of Sensitive Materials

**Figure 2:**
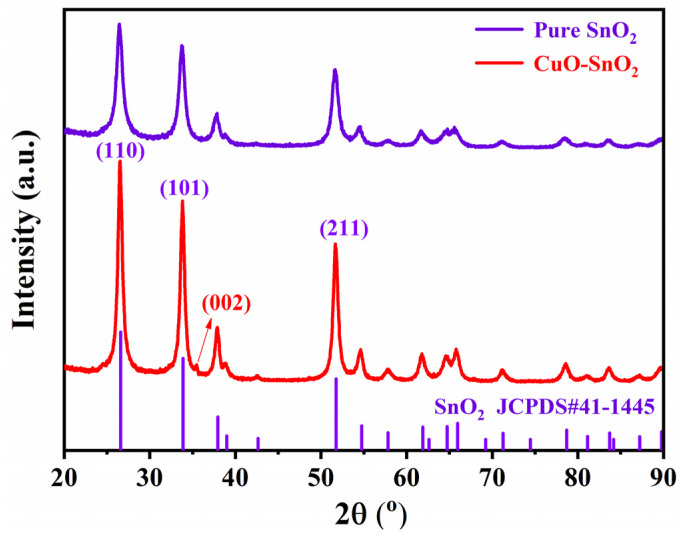
XRD patterns for pure SnO_2_ and 1% CuO-SnO_2_ samples.

To unveil the structural features of the obtained sensitive materials, XRD was first employed to explore the crystalline information of pure SnO_2_ and 1% CuO-SnO_2_ samples in Figure 2. In terms of pure SnO_2_, the peaks at 26.5, 33.6, and 51.7° were, respectively, indexed into (110), (101), and (211) crystal planes of tetragonal SnO_2_ (JCPDS#41-1445), consistent with our previous work [20,21]. In regard to the 1% CuO-SnO_2_ sample, besides the existence of feature peaks pertaining to the SnO_2_ component, another minor reflection peak at 35.3° was assigned to the crystal plane (002) of CuO (JCPDS #48-1584) [22]. The weak peak intensity was primarily originated from the low CuO content. The feature peaks of SnO_2_ material did not shift after CuO incorporation, reflecting an unvaried crystal structure. These XRD patterns were well in accordance with expectations, indicating a successful synthesis of target materials through the proposed method.

**Figure 3:**
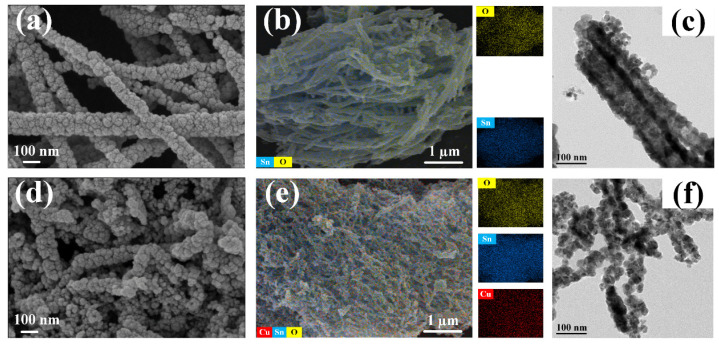
(**a**,**d**) SEM images, (**b**,**e**) element mapping, and (**c**,**f**) TEM images of pure SnO_2_ and CuO-SnO_2_ samples.

SEM images were then adopted to unveil the morphology characteristics, as displayed in Figure 3a,d. Obviously, both samples exhibited 1D nanotube-like structures assembled by multiple nanoparticles, which was beneficial for molecular gas adsorption due to the large surface area as well as charge carrier transport arising from constrained scattering within reduced dimensions. During the calcination treatment, PVP was decomposed into some gaseous intermediates that promoted a hollow nanotube structure [23]. Interestingly, 1% CuO-SnO_2_ nanotubes showed shorter length and fluffier skeletons than pure SnO_2_ counterparts, probably imparting richer adsorption sites and a wider mass transfer space during the gas-sensing tests for an enlarged sensitivity and accelerated reaction kinetic. The element mapping in Figure 3b,e showed the co-existence of Sn, O, and Cu elements within 1% CuO-SnO_2_, as well as of Sn and O elements within pure SnO_2_. The uniform and similar element distribution verified the intimate interactions between CuO and SnO_2_ components. The TEM images in Figure 3c,f further confirmed the hollow nanotube-like structure of both samples.

**Figure 4:**
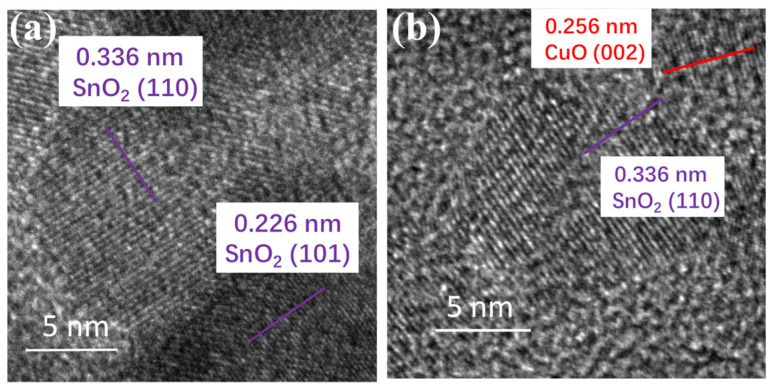
**HRTEM images** of (**a**) pure SnO_2_ and (**b**) 1% CuO-SnO_2_ samples.

To verify the existence of heterojunctions within the 1% CuO-SnO_2_ composites, we further investigated the high-resolution TEM (HRTEM) images for both samples in Figure 4. As shown in Figure 4a, the crystal planes (110) and (101) appeared for pure SnO_2_, corresponding to the lattice spacings of 0.336 and 0.226 nm, respectively. In regard to the CuO-SnO_2_ composites in Figure 4b, the interwoven crystal planes of (110) for SnO_2_ and (002) for CuO existed with relevant lattice spacings of 0.336 and 0.256 nm, which strongly verified the production of heterojunctions between SnO_2_ and CuO components.

### 3.2. Gas-Sensing Performance

When investigating the gas-sensing performance, it was found that a pure SnO_2_ sensor showed a negligible response (~1.3) and large noise background for 20 ppm H_2_S at room temperature, and no signal below this concentration (Supplementary Material, Figure S1a). Inspiringly, the sensor response was significantly boosted after CuO incorporation, suggesting a remarkable sensitization effect through the p–n heterojunctions (Supplementary Material, Figure S1b). In addition, the 1% CuO-SnO_2_ sensor exhibited the largest response among all prepared devices, revealing an important impact of the componential ratio on the sensing performance. Subsequently, four LED light sources (power density of 660 mW/cm^2^) including blue light (465 nm), green light (520 nm), yellow light (590 nm), and red light (620 nm), installed above the sensors at a distance of 1.5 cm were employed to unveil the photoelectric effect on the sensor performance. Even under blue light illumination, the pure SnO_2_ sensor did not show any signal toward 10 ppm H_2_S owing to the smaller light energy (2.67 eV) relative to the bandgap (3.6 eV) of SnO_2_ material being insufficient to activate the electrons from the valence band to conduction band (Supplementary Material, Figure S1c). We also prepared pure CuO sensors (Supplementary Material, Figure S2) and investigated the sensor performance for 10 ppm H_2_S at room temperature under different lighting conditions (dark, red, yellow, blue, green). The results showed that the baseline resistance of the pure CuO sensor exceeded the measurable limit of our laboratory setups. That is because few conducting pathways were constructed under all cases different from the continuous film displayed in a previous work [24], which could be observed from Figure S2. Therefore, the primary sensing element was SnO_2_, while CuO served in a secondary role within the CuO-SnO_2_ heterojunctions.

**Figure 5:**
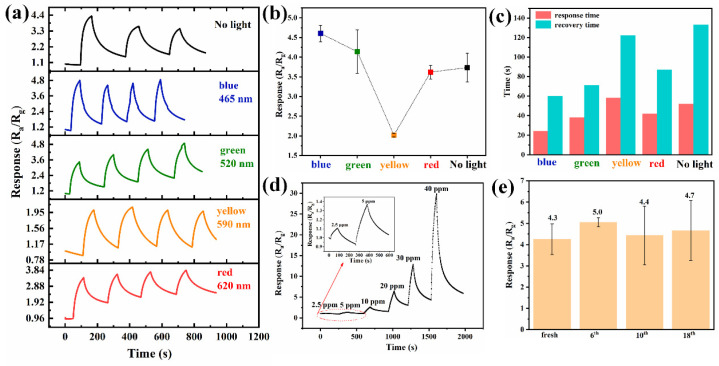
The performance of the 1% CuO-SnO_2_ sensor toward H_2_S gas at room temperature: (**a**) real-time response, (**b**) mean response, and (**c**) response and recovery times for 10 ppm H_2_S under different visible light activations, as well as (**d**) dynamic response as function of H_2_S concentration and (**e**) long-term stability with 10 ppm H_2_S under blue light illumination.

With respect to the 1% CuO-SnO_2_ sensor shown in Figure 5a, it was found that light irradiation mitigated the baseline drift as compared to in the dark case, primarily due to the light-enhanced molecules unbinding from the high-energy sorption sites on the material surface. Among all illumination conditions, the response under yellow light was the least effective. We assumed that light irradiation played a dual role of photo-activating the semiconductors and detaching the adsorbed gas molecules during the gas-sensing tests. Despite the generation of photo-activated electron-hole pairs within the sensing layer, simultaneously, the dynamic adsorption/desorption balance shifted to a predominant desorption process under yellow light. These two behaviors compromised the gas–solid interaction and reduced the sensor response. In addition, blue light brought about the largest response (4.7) and quickest response/recovery speeds (21/61 s) for 10 ppm H_2_S with nice repeatability, as exhibited in Figure 5b,c. Also, the sensor could detect H_2_S gas with a concentration ranging from 2.5 to 40 ppm with a LoD value of 2.5 ppm, as displayed in Figure 5d, manifesting a wide detection range and a trace recognition ability. In Figure 5e, the 1% CuO-SnO_2_ sensor delivered good long-term stability for 10 ppm H_2_S with 9% fluctuation in the mean response over 18 days.

Recent work about SnO_2_-based H_2_S sensing was compared with this work in Table 1. Obviously, the overwhelming majority of previous works were conducted at high operation temperatures (OT ≥ 150 °C) [25,26,27,28,29,30]. Even under a heating mode, the sensor response was still weak [25,26,29]. Taking into account the response, response/recovery times, (T_res_/T_rec_) and OT, the 1% CuO-SnO_2_ sensor in this work showcased competitive performance.

**Figure 6:**
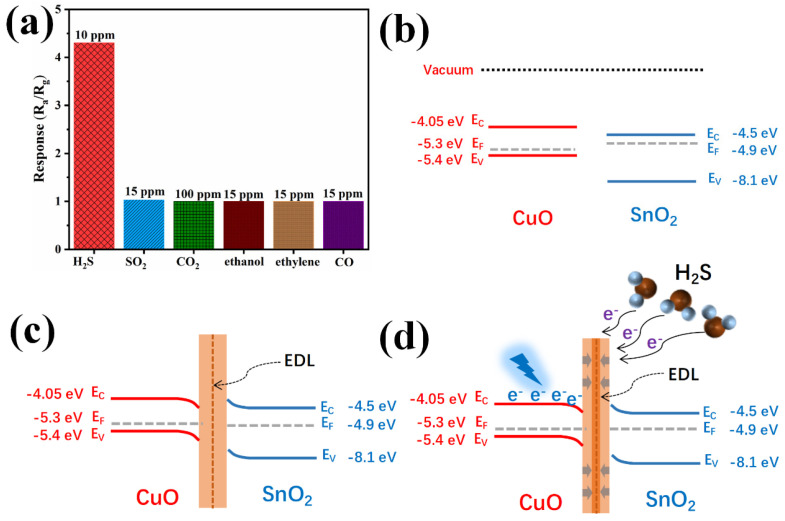
(**a**) The cross-sensitivity of the 1% CuO−SnO_2_ sensor toward different gasses under blue light activation, the energy level relationship (**b**) before and (**c**) after intimate contact between both components, and (**d**) the band diagram of CuO−SnO_2_ heterojunctions after H_2_S adsorption under blue light activation.

Afterward, the sensor response toward different interfering gases with larger concentration including SO_2_ (15 ppm), CO_2_ (100 ppm), ethanol (15 ppm), ethylene (15 ppm), and CO (15 ppm) than H_2_S case (10 ppm) was revealed in Figure 6a. The much larger response toward H_2_S gas than that toward the rest demonstrated an excellent H_2_S selectivity.

### 3.3. Sensing Mechanism Analysis

According to previous work [31,32], the energy level relationship was established as shown in Figure 6b. Due to the difference in the Fermi level of p-type CuO and n-type SnO_2_, the electrons flow from SnO_2_ to CuO upon an intimate contact, which mainly produces an electron depletion layer (EDL) on the SnO_2_ surface, as depicted in Figure 6c. Here, the CuO content was so low that the overall semiconducting polarity of the composites was the same as that of pure SnO_2_. Under dark conditions, pure SnO_2_ was insensitive to H_2_S gas at room temperature. Noteworthily, the incorporation of CuO evidently boosted the sensor response as the abundant interfaces generated between these p–n heterojunctions were favorable for more molecular adsorption. Upon H_2_S injection, as displayed in Figure 6d, reducing H_2_S molecules donated electrons to n-type SnO_2_ by reacting with oxygen species on the SnO_2_ surface, as expressed in Equations (1)–(3), contracted the EDL, and then lowered the sensor resistance. After air purification, H_2_S molecules were desorbed from the sensing layer with the withdrawing of these electrons, reversibly restoring the resistance.

O_2_ (gas) → O_2_ (ads) (1)

O_2_ (ads) + e^−^ → O_2_^−^ (ads) (2)

2H_2_S (g) + 3O_2_^−^ (ads) → 2SO_2_ (g) + 2H_2_O (g) + 3e^−^ (3)

*hv* → h^+^ (*hv*) + e^−^ (*hv*) (4)

O_2_ (ads) + e^−^ (*hv*) → O_2_^−^ (*hv*) (5)

2H_2_S (g) + 3O_2_^−^ (*hv*) → 2SO_2_ (g) + 2H_2_O+ 3e^−^ (6)

The enhanced response under blue light activation was ascribed to the following factors. As mentioned before, the bandgap of the SnO_2_ material exceeded the light energy, and no photoelectric effect occurred. Worse still, light activation readily detached the adsorbed H_2_S molecules and attenuated the response with respect to the dark conditions. In regard to CuO-modified SnO_2_ nanotubes, on the one hand, rich heterojunctions strengthened the gas–solid interactions, simultaneously producing a built-in electric field pointing to the CuO side. On the other hand, the smaller bandgap of CuO material (1.35 eV) relative to the light energy favored a significant photo-activation behavior. That is to say, blue light activated the transition of electrons from the valence band of the CuO to the conduction band, as shown in Equation (4). Therefore, the additional photo-generated electrons were transferred to the SnO_2_ side under the effect of the built-in field. As these electrons were more active than the inherent ones, more ambient oxygen molecules were readily ionized and then reacted with H_2_S molecules, as indicated in Equations (5) and (6). All these aspects collectively contributed to a much stronger response from the 1% CuO-SnO_2_ sensor than for that of the pure SnO_2_ counterpart under dark and light conditions. As for the light-accelerated reaction speeds (response and recovery times), reduced binding force between adsorbed gas molecules and sorption sites as well as a quickened achievement of the dynamic adsorption/desorption equilibrium could account for this.

## 4. Conclusions

In conclusion, hollow CuO-SnO_2_ nanotubes were prepared by electrospinning technology for room-temperature trace H_2_S detection under blue light activation in this work. After componential optimization, a 1% CuO-SnO_2_ sensor delivered a large response of 4.7 and quick response/recovery speeds of 21/61 s for 10 ppm H_2_S, as well as an LoD value of 2.5 ppm. Moreover, favorable repeatability, long-term stability, and selectivity were demonstrated. Combined strategies involving 1D nanostructures, p–n heterojunctions, and photoelectric effects pave the way for the subtle design of future optoelectronic devices with the merits of boosted sensitivity, low power consumption, and high miniaturization.

## Figures and Tables

**Table 1:** Recent work about H_2_S-sensing performance of SnO_2_-based gas sensors.

Materials	Response	T_res_/T_rec_ (s)	OT (°C)	Ref.
Cu/SnO_2_	275%@300 ppm	15/95	150	[25]
W/SnO_2_	360%@10 ppm	17/7	260	[26]
Au/SnO_2_	3400%@5 ppm	35/-	300	[27]
ZnO/SnO_2_	1500%@5 ppm	-/-	260.6	[28]
CuO-SnO_2_ nanofibers	85.71%@50 ppm	100/109	200	[29]
Pt/SnO_2_ nanotubes	8930%@1 ppm	99.5/111.6	300	[30]
1% CuO-SnO_2_ nanotubes	470%@10 ppm	21/61	25	this work

## Data Availability

All relevant data are within the paper.

## Supplementary Materials

The following supporting information can be downloaded at https://www.mdpi.com/article/10.3390/s24196420/s1: the test procedures, characterization techniques, Figure S1: The dynamic response of pure SnO_2_ sensor and all SnO_2_-based sensors toward 20 ppm H_2_S under dark condition at room temperature, and the real-time response of pure SnO_2_ sensor toward 10 ppm H_2_S under blue light activation at room temperature. Figure S2: The real images of pure CuO sensors.
