# Supplementary material for: Photoelectric H2S Sensing Based on Electrospun Hollow CuO-SnO2 Nanotubes at Room Temperature
Source: Sensors (Basel). 2024 Oct 3;24(19):6420. doi: 10.3390/s24196420 (PMC11479242; doi:10.3390/s24196420)
Supplement: Supplementary file 1 [file sensors-24-06420-s001.zip › sensors-3224034-supplementary.pdf]

sensors

MDPI

Supplementary Materials

Photoelectric H_2S Sensing based on Electrospun Hollow CuO-SnO_2 Nanotubes at Room Temperature

Cheng Zou [2,3], Cheng Peng [2], Xiaopeng She [2], Mengqing Wang [2], Bo Peng [1,*] and Yong Zhou [2,*]

[1] Liangjiang School of Artificial Intelligence of Chongqing University of Technology, Chongqing, 401135, People's Republic of China
[2] Key Laboratory of Optoelectronic Technology and System of Ministry of Education, College of Optoelectronic Engineering, Chongqing University, Chongqing 400044, People's Republic of China
[3] Chongqing Key Laboratory of Natural Product Synthesis and Drug Research, Innovative Drug Research Center, School of Pharmaceutical Sciences, Chongqing University, Chongqing 401331, People's Republic of China
* Correspondence: pengbo86@126.com; zhyf@cqu.edu.cn;

Test Procedures

During the dynamic gas-sensing tests, the gas concentration of CO_2, ethanol, ethylene, CO, SO_2 and H_2S was calculated by $(C_sF_s)/(F_s+F_c)$, where C_s denoted the standard target gas concentration in the commercial certificated steel bottle while F_s and F_c respectively termed the flow rate of the standard target gas and carrier gas (dry air). The sensor response was separately calculated using R_a/R_g and R_g/R_a toward reducing and oxidizing gases, wherein R_a and R_g represented the stable sensor resistance in the background environment and target gas, respectively. All tests were carried out in dry air (0.3% RH) unless otherwise stated. The response/recovery time (T_{res}/T_{rec}) was defined as the elapsed time reaching 90% and 10% of the maximum response upon target gas injection and air purification, respectively.

Characterization Techniques

The crystal phases of the as-prepared materials were investigated by Panalytical Empyrean XRD diffractometer (Netherlands) using a CuKα radiation source with a scanning rate at 5°/min. The morphological features of the samples were analyzed by a field emission scanning electron microscope (FESEM, S-4800, HITACHI, Japan) performed at an acceleration voltage of 5 kV. Transmission electron microscopic (TEM) images were gathered on a JEOL-2010 TEM manipulated at 200 kV.

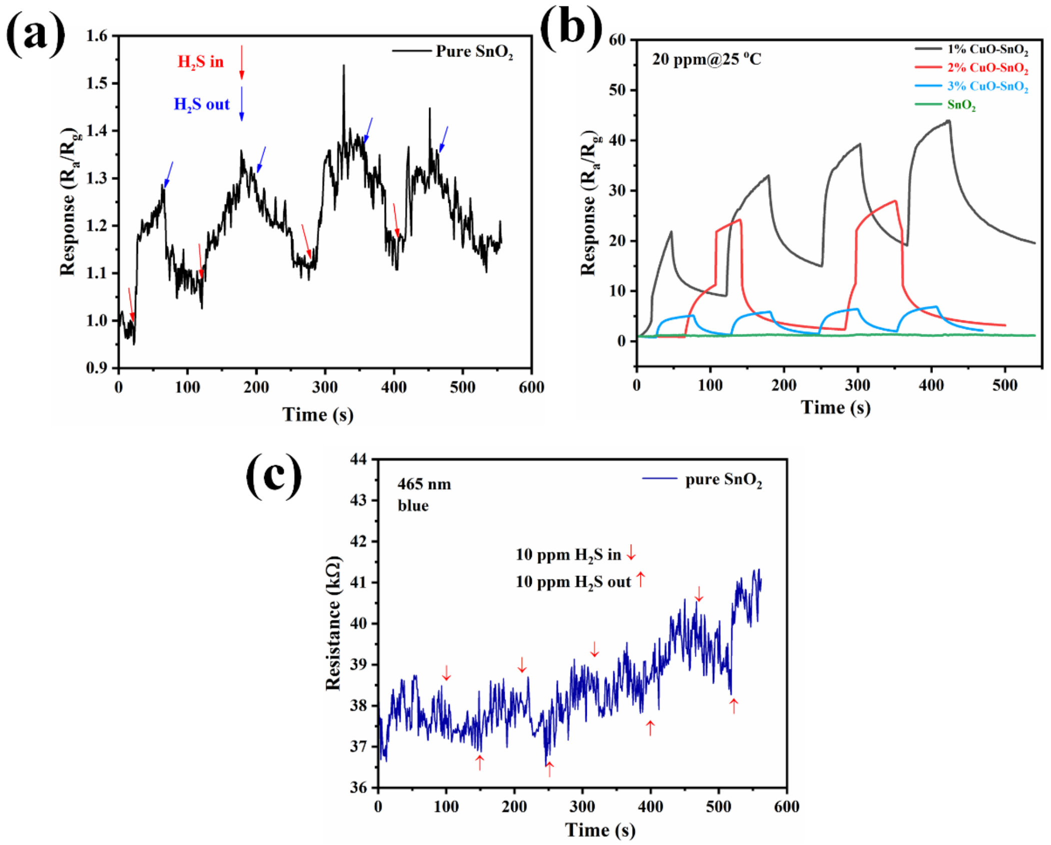

Figure S1. The dynamic response of (a) pure SnO_2 sensor and (b) all SnO_2-based sensors toward 20 ppm H_2S under dark condition at room temperature, and (c) the real-time response of pure SnO_2 sensor toward 10 ppm H_2S under blue light activation at room temperature.

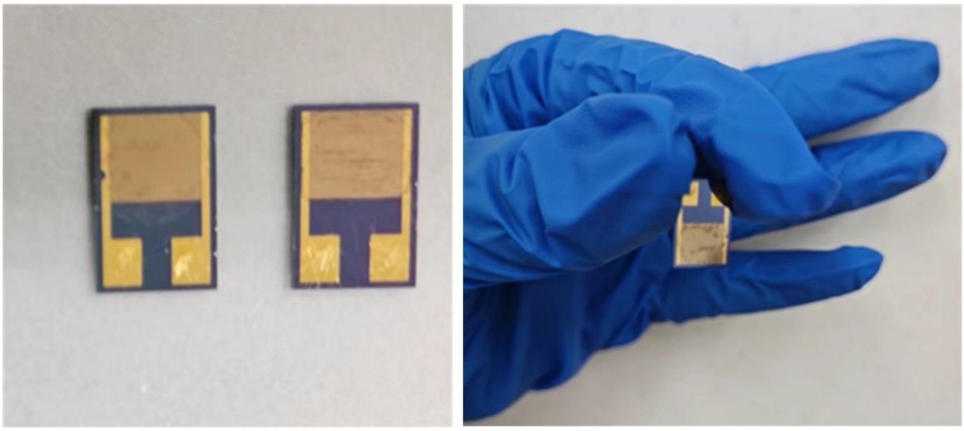

Figure S2. The real images of pure CuO sensors.
